# Insane Asylums

**Published:** 1898-05

**Authors:** 


### Insane Asylums.

Dr. S. Weir Mitchell contends that the handsome institutions erected at a great expense for the treatment of the insane, as a rule, fail to accomplish the results for which they were designed. He thinks that many more insane patients should be cured. He states a belief that the asylums are crippled by politics; that there is a lack of skilled nurses. No unskilled or ignorant person should come in contact with the patients. He advocates "the abolition of the immense prisons for the insane, with their contingent of ignorant political wardens, keepers and turnkeys, and the substitution of hospitals for the mentally diseased, without walls and barred gates, with separate small houses, each with its attendants and abundant appliances for work and play." —*Pacific Medical Journal.*

